# Impact of Diabetes Mellitus on the Presentation and Response to Treatment of Adults With Pulmonary Tuberculosis in Qatar

**DOI:** 10.1093/ofid/ofy335

**Published:** 2018-12-19

**Authors:** Khalid M Dousa, Abdelrahman Hamad, Mohamed Albirair, Hussam Al Soub, Abdel-Naser Elzouki, Mahmoud I Alwakeel, Bonnie A Thiel, John L Johnson

**Affiliations:** 1 Division of Infectious Diseases & HIV Medicine, Case Western Reserve University, University Hospitals Cleveland Medical Center, Cleveland, Ohio; 2 Department of Internal Medicine, Weill Cornell Medical Collage, Hamad Medical Corporation, Doha, Qatar; 3 Department of Global Health, University of Washington, Seattle, Washington; 4 Tuberculosis Research Unit, Department of Medicine, Case Western Reserve University and University Hospitals Cleveland Medical Center, Cleveland, Ohio

**Keywords:** diabetes mellitus, migrant workers, pulmonary tuberculosis

## Abstract

**Background:**

Persons with diabetes mellitus (DM) have a 3-fold increased risk of tuberculosis (TB). Atypical radiographic findings and differences in bacteriologic response during anti-TB treatment have been reported in earlier studies; however, the findings have varied. We evaluated the effect of DM on manifestations and response to treatment in adults with pulmonary TB in Qatar.

**Methods:**

The impact of DM on the clinical and radiographic presentations of pulmonary TB and bacteriologic response during anti-TB treatment was evaluated between January 2007 and December 2011, comparing patients with and without DM. This is a retrospective unmatched case-control study conducted at a large national hospital. Cases and controls were randomly selected from patients diagnosed with pulmonary TB over a 5-year period. Sputum culture conversion was assessed after 2 months of anti-TB treatment.

**Results:**

Clinical symptoms were similar between patients with and without DM. Patients with DM had a higher initial sputum acid-fast bacillus (AFB) smear grade and were less likely to have cavitary lesions on initial chest radiographs than patients without DM. Of 134 adults with DM and TB, 71 (53%) remained sputum culture positive after 2 months of anti-TB treatment, compared with 36 (27%) patients without DM.

**Conclusions:**

DM was associated with atypical radiographic findings and delayed sputum culture conversion at 2 months in adults with pulmonary TB in Qatar. Increased health education of patients with DM about symptoms of TB, low thresholds for evaluation for active TB, and close monitoring of bacteriologic response to treatment among patients with TB and DM are warranted.

Persons with diabetes mellitus (DM) have a 3-fold increased risk of developing tuberculosis [1]. This increased risk has been ascribed to the intracellular nature of tuberculosis (TB) infection and impaired T-cell-mediated immune response in persons with DM [2]. Increasing rates of obesity and metabolic syndrome have led to an increasing prevalence of DM in many regions of the world.

Earlier studies of the impact of DM on clinical, radiographic, and bacteriologic presentations of TB have suggested that patients with DM are more likely to present with atypical chest radiographic features, including more frequent lower lobe disease, fewer cavitary lesions, and diffuse lung involvement; however, reports have been inconsistent [3–6]. Two recent studies have shown that atypical chest radiographic findings and more severe radiographic disease were associated with higher hemoglobin A1c levels and poor chronic glycemic control among persons with DM and TB [7, 8].

Response to anti-TB treatment has been reported to be slower and associated with worse outcomes in patients with DM and pulmonary TB. Delayed sputum smear and culture conversion after 2 months of therapy [9, 10] and increased treatment failure [11, 12], relapse, and death in patients with DM being treated for TB have been described; however, other studies found no differences in bacteriologic response and TB treatment outcomes comparing persons with and without DM [13, 14]. The degree of dysglycemia may also affect treatment response. In a prospective hospital-based study from India, patients with pulmonary TB and DM with initial poor glycemic control had higher sputum smear grade, delayed smear conversion after 2 months of treatment, and higher treatment failure and relapse rates [15].

Few studies have focused on the impact of DM on the clinical presentation of TB and response to TB treatment in the Middle East. Tuberculosis is a major public health problem in Middle Eastern countries [16]. Qatar is an oil-rich nation on the Arabian Peninsula with a population of 2.5 million people. The incidence of TB in 2015 was 34 per 100 000 population per year [17]. Approximately 75% of the population is foreign-born, and, in a 2015 abstract, 90% of new cases of TB occurred in foreign-born persons [18]. In this study, we describe the impact of DM on the initial clinical, radiographic, and bacteriological characteristic of patients with pulmonary TB and their bacteriologic response to anti-TB treatment in Qatar.

## METHODS

### Study Design and Patient Population

The study is a retrospective unmatched case-control study conducted at Hamad General Hospital (HGH). HGH is a 603-bed public hospital in Doha, Qatar, and is the largest hospital in the country. This hospital provides inpatient and outpatient tertiary care in medicine and surgery for the residents of Qatar (nationals and expatriates), making it a good center for population-based studies [19]. Between January 2011 and January 2015, adults who were 18 years and older with pulmonary TB were eligible and were considered for this study. Based on prior studies, differences in sputum culture conversion rates comparing pulmonary TB patients with and without DM have varied from none to up to 15% [9–14]. Under the assumption of a 15% difference in event rate of culture conversion between pulmonary TB patients with or without DM, we determined that inclusion of 134 patients in each group would be sufficient to provide a power of 80% to detect a difference in culture conversion, with a 2-sided confidence interval of 90%. We identified 2794 adults with the diagnosis of pulmonary TB from our hospital TB registry. Within this population, 340 adults had both pulmonary TB and DM. Of these patients, 134 were randomly sampled as cases. A control group of 134 patients without DM was randomly sampled from the population set of 2794 TB patients. Exclusion criteria were HIV co-infection, end-stage renal disease, and the use of immunosuppressive therapy or tumor necrosis factor–alpha inhibitors. The study was reviewed by the institutional review board at Hamad Medical Corporation Research Center and was deemed exempt under Supreme Council of Health guidelines.

Pulmonary TB was diagnosed based on symptoms, chest radiographic findings, and sputum acid-fast bacillus (AFB) smear microscopy with culture confirmation. Smear microscopy was performed by the Ziehl-Neelsen method; Smears were graded using the World Health Organization/International Union Against Tuberculosis and Lung Disease scheme [20]. Cultures were done using Lowenstein-Jensen media. Patients were initially hospitalized to begin anti-TB treatment. Tuberculosis was treated with 2 months of oral rifampicin 10 mg/kg/d, isoniazid 5 mg/kg/d, pyrazinamide 25 mg/kg/d, and ethambutol 20 mg/kg/d, followed by 4 months of daily isoniazid and rifampicin according to World Health Organization guidelines [21]. Sputum AFB smear testing was repeated after 2 weeks and then weekly until negative to monitor response to treatment and infectiousness. Patients were subsequently discharged to complete treatment on an ambulatory outpatient basis. Sputum culture was repeated after 2 months of anti-TB treatment. Diagnosis of DM was based on serum hemoglobin (Hb) A1c >6.5%, according to published criteria of the American Diabetes Association [22]. DM was considered poorly controlled if HbA1c was >8% [23, 24].

### Patient Characterization and Smear Conversion Follow-up

The Hamad General Hospital TB registry was used to identify patients by medical record numbers. Data were collected using a standardized data abstraction form developed for the study. Data regarding demographics, symptoms, radiographic findings, sputum acid-fast smear grade and conversion, sputum culture results, adverse events, and DM control, as indicated by HbA1c level, were recorded. Primary outcomes of the study were differences in the clinical, radiographic, and bacteriologic presentation of pulmonary TB and bacteriologic response during treatment (sputum culture conversion to negative after 2 months of anti-TB treatment) comparing patients with and without DM. Secondary objectives included assessment of the impact of glycemic control, as indicated by the HbA1c, on these outcomes. Radiological findings and laboratory values were collected by review of the patients’ electronic medical records. Chest radiographs were read by 2 experienced radiologists who recorded the presence or absence of cavitation and laterality of lesions. Unilateral lung involvement was further classified as upper lobe, lower lobe, and diffuse disease.

### Data Analysis

We compared findings among TB patients with DM with those without DM. For bivariate analyses, we used Pearson’s χ^2^ test to compare categorical frequencies, the Student *t* test to compare means for normally distributed continuous variables, and the Mann-Whitney *U* test for non–normally distributed data. We performed univariate analysis of factors associated with having DM and then used a logistic regression model to assess the additive effects of variables that were significant in the univariate analysis. We assigned statistical significance as a *P* value of less than .05 (2-tailed) and performed statistical analysis using R, version 3.5.1 (July 2, 2018 release).

## RESULTS

### Baseline Clinical Characteristics

Table 1 shows the pretreatment demographic and clinical characteristics of the study population. Most were males. Ninety-three percent of the patients with TB and DM and 97% of the patients with TB and without DM were foreign-born. Eighty-five percent of the cases and 88% of the controls were migrant workers. Qatar-born patients constituted only 4% of the study population. Patients with TB and DM were more than 10 years older on average than those with TB only (*P* < .0001). The mean body mass index (BMI) at the time of diagnosis of TB was greater among TB patients with DM compared with those without DM (22.4 and 20.8, respectively; *P* = .0003). Lower lobe involvement on chest radiograph occurred more frequently among patients without DM (*P* = .07). Similarly, cavitary lesions also occurred more often in patients without DM (*P* = .007). The pretreatment sputum AFB smear grade, a measure of sputum bacillary load, was higher among patients with DM compared with nondiabetic patients (*P* = .02).

**Table 1.** Pretreatment Characteristics of Patients With Pulmonary TB With or Without Diabetes Mellitus in Qatar, 2007–2011

	TB Without DM (n = 134), No. (%)	TB With DM (n = 134), No. (%)	Univariate *P* Value	Multivariable *P* Value, aOR (95% CI)^a^
Pretreatment				
Age, mean ± SD, y	33.5 ± 9.2	47.5 ± 10.2	<.0001	0.0001, 1.14 (1.10–1.17)
Male sex	107 (80)	114 (85)	.33	
Weight, mean ± SD, kg	55.8 ± 9.3	60.1 ± 11.3	.0003	
BMI, mean ± SD, kg/m^2^	20.8 ± 3.2	22.4 ± 4.2	.0003	0.15, 1.06 (0.98–1.17)
Foreign-born	130 (97)	125 (93)	.25	
Migrant worker	118 (88)	114 (85)	.59	
Years residing in Qatar, mean ± SD	1.72 ± 0.57	1.85 ± 0.36	.02	0.33, 1.38 (0.71–2.65)
Contact with TB patient	13 (10)	12 (9)	1.0	
Symptoms				
Fever	94 (70)	99 (74)	.58	
Weight loss	68 (51)	63 (47)	.62	
Cough	112 (84)	122 (91)	.10	
Hemoptysis	32 (24)	38 (28)	.49	
Chest radiographic and CT findings			.41	
Right lung disease	48 (36)	53 (40)		
Left lung disease	41 (31)	46 (34)		
Bilateral disease	45 (34)	35 (26)		
Lobar involvement			Reference	
Upper lobe lesions	52 (39)	49 (37)		
Lower lobe lesions	34 (25)	38 (28)		
Diffuse disease (>1 lobe)	48 (36)	47 (35)		
Cavitary disease	80 (60)	57 (43)	.007	0.04, 0.52 (0.28–0.96)
Sputum smear grade			.02	
Negative	34 (25)	36 (27)		Reference
1+	18 (13)	4 (3)		0.06, 0.26 (0.05–0.98)
2+	11 (8)	12 (9)		0.65, 0.75 (0.22–2.61)
3+	71 (53)	82 (61)		0.87, 1.06 (0.52–2.16)
Drug side effects			.53	
Hepatitis	16 (12)	14 (10)		
Others	6 (4)	3 (2)		
No side effect	112 (84)	117 (87)		
Drug susceptibility to isoniazid and rifampicin	131 (98)	130 (97)	1.00	

^a^Multiple logistic regression analysis modeling the probability of having TB with DM vs TB with no DM.

Abbreviations: aOR, adjusted odds ratio (exponentiated coefficient); BMI, body mass index; CI, confidence interval; CT, computed tomography; DM, diabetes mellitus; TB, tuberculosis.

A logistic regression model was used to jointly assess the effects of the significantly associated variables (except weight, which was highly correlated with BMI) on the probability of having TB with DM. In Table 1, the last column shows the adjusted *P* values and odds ratios for the multivariable model. Age and lung cavitary disease remained significant predictors of having DM with TB; the probability of having DM with TB (relative to TB without DM) increased with age and cavitary lung disease in this population. The odds ratio for age effect was 1.14, with a 95% confidence interval (CI) of 1.10–1.17; the odds ratio for lung cavitary disease effect was 0.52, with a 95% CI of 0.28–0.96.

### Glycemic Control

The mean HbA1c among patients with TB and DM was 10.7%, with a standard deviation of 2.2%. Sixty-two percent of patients with DM were on treatment with oral hypoglycemic medications at the time of TB diagnosis. Nine percent were on insulin, and 32% were not on medication for DM. We also examined the association between the level of dysglycemia among diabetics and the clinical and radiological manifestations of TB. In both univariate and logistic regression models, the clinical, radiographic, and bacteriological findings at the time of diagnosis of TB did not differ according to the degree of dysglycemia (Hgb A1c ≤8% or >8%) (Table 2).

**Table 2.** Impact of Pretreatment Glycemic Status on the Chest Radiographic and Bacteriologic Presentation of TB and 8-Week Sputum Culture Conversion in Patients With Pulmonary TB and DM in Qatar, 2007–2011

	HbA1c ≤ 8% (n = 64), No. (%)	HbA1c > 8% (n = 70), No. (%)	*P* Value
Chest radiographic and CT findings			.16
Right lung disease	24 (37)	29 (40)	
Left lung disease	18 (29)	25 (36)	
Bilateral disease	22 (34)	16 (23)	
Lobar involvement			.1
Typical, upper lobe	24 (37)	37 (52)	
Atypical, lower lobe, and diffuse	40 (63)	33 (48)	
Sputum smear grade			.07
Negative	19 (29)	19 (27)	
1+	7 (10)	2 (3)	
2+ and 3+	38 (61)	49 (70)	
Culture conversion to negative during the first 2 mo of anti-TB treatment	45 (70)	32 (46)	.09

Abbreviations: CT, computed tomography; DM, diabetes mellitus; HbA1c, hemoglobin A1c; TB, tuberculosis.

### Response During Anti-TB Treatment

Of 134 TB patients with DM, 63 (47%) converted their sputum culture to negative after 2 months of anti-TB treatment, compared with 98 (73%) patients without DM (*P* < .0001). To adjust for covariates, we modeled the probability of having culture conversion at 2 months by DM plus age, which was an independently associated risk factor. Inclusion of both age and DM in the multivariable model resulted in DM remaining the only significant factor. The odds ratio for delayed conversion (culture conversion to negative after completing 2 months of anti-TB therapy) given DM relative to late conversion without DM was 2.3, with a 95% CI of 1.25–4.35. Culture conversion did not differ between patients with severe hyperglycemia at diagnosis and those with lesser degrees of dysglycemia. The frequency of adverse drug reactions during anti-TB treatment also did not differ between patients with and without DM (Table 1).

## DISCUSSION

Diabetes mellitus is a significant burden for persons infected with TB [25]. DM increases the risk of developing TB by 3-fold [26]. The prevalence of DM and obesity is increasing worldwide, with an estimated rise from 415 million to 642 million over the next 2 decades. In Qatar, one-half of native Qataris are obese, and 17% have DM [27]. Population modeling studies have demonstrated that the interaction of DM, obesity, and TB now accounts for a substantial portion of new TB cases in low– and moderate–TB incidence countries [25]. Since 2010, Qatar has experienced an influx of several hundred thousand migrant workers employed in construction work for the 2022 football World Cup. More than 85% of patients with TB in this study were migrant workers. These workers are in daily contact with the general population, who are considered at higher risk of acquiring TB based on the high prevalence of DM in the country.

The most common presenting symptoms of TB among the patients in this study were cough, fever, and weight loss. Symptoms of cough, fever, weight loss, and hemoptysis did not differ between patients with and without DM, which was consistent with earlier studies [28]. Some reports suggested a more severe onset of disease [26, 29].

The radiographic presentation of pulmonary TB in patients with DM in Qatar was atypical. We observed less frequent cavitary disease in patients with DM. Several studies showed similar trends [3]; however, other studies showed no difference in radiographic findings between patients with and without DM [30, 31]. In 2014, Chiang et al. examined the radiological findings of 581 culture-positive pulmonary TB patients and concluded that lower lung infiltrates, extensive parenchymal lung lesions, and multiple pulmonary cavities occurred more frequently among patients with TB and DM, especially among those with poor glycemic control [7]. In a univariate analysis, we observed no relationship between glycemic control and radiological presentation, which contrasts with the study by Huang et al. from China, which showed that unusual and atypical radiological patterns occurred more frequently in persons with glycosylated hemoglobin >8% [7].

 Monitoring sputum smears and cultures during anti-TB treatment is important to assess the patient’s response to therapy. The time until conversion of cultures to negative during anti-TB treatment is used as a predictor of treatment success [32]. DM has been associated with delayed sputum conversion and TB treatment failure [29, 30, 33]. We observed a higher initial sputum smear grade among patients with TB and DM and found that delayed sputum culture conversion after 2 months of anti-TB treatment occurred more frequently in patients with TB and DM. No association between initial glycemic status and culture conversion was identified in our study, which was consistent with the retrospective analysis of Martínez-Oceguera et al., who examined 88 sputum culture results from patients with DM and found no significant impact of glycemic control on culture conversion [34]. In a logistic regression model to examine this association, we found a trend toward low odds of culture conversion by 2% for every 1% increase in serum HbA1c; however, this observation did not reach statistical significance, possibly due to our sample size and limited power to detect a significant difference. Further studies to examine this association may be warranted.

This study is the first to examine the association of TB and DM in Qatar. Our study has several limitations. It is a retrospective chart review from a single center. Some patients with DM and TB may have been overlooked before the hospital implemented an electronic medical record system in 2009. The number of cases of TB and DM in Qatar was moderate, and we had limited statistical power to detect differences between patients with TB and DM in the analysis of chest radiographic findings and glycemic control. The strengths of our study include its conduct at the largest hospital in the country with the widest catchment area, performance of hemoglobin A1c and sputum AFB smears and cultures at a single quality-controlled laboratory, and central reading of chest radiographs by a single group of experienced radiologists.

## CONCLUSIONS

DM was associated with atypical radiological findings in adults presenting with pulmonary TB in Qatar, most of whom were migrant workers, but had no impact on other presenting clinical features of pulmonary TB. Sputum culture conversion to negative after 2 months of anti-TB treatment was delayed in patients with DM. Increased health education of patients with DM about symptoms of TB, low thresholds for evaluation for active TB, and close monitoring of bacteriologic response to anti-TB treatment among patients with TB and DM may be warranted.
